# Opium tincture for opioid substitution treatment

**DOI:** 10.1192/j.eurpsy.2021.455

**Published:** 2021-08-13

**Authors:** M. Nikoo, K. Kianpoor, N. Nikoo, S. Javidanbardan, A. Kazemi, F. Choi, M. Vogel, A. Gholami, S. Tavakoli, J. Wong, E. Moazen‐Zadeh, R. Givaki, M. Jazani, F. Mohammadian, N. Markazi Moghaddam, C. Schütz, K. Jang, S. Akhondzadeh

**Affiliations:** 1 Psychiatry, Addictions and Concurrent Disorders Research Group, Institute of Mental Health, Vancouver, Canada; 2 Division Of Substance Use Disorders, Psychiatric Services of Thurgovia, Thurgovia, Switzerland; 3 Substance Use Disorder, Kian Methadone Maintenance Treatment Clinic, Sari, Iran; 4 Private Practice, Rooz-e-No, Methadone Maintenance Treatment Clinic, Shiraz, Iran; 5 Department Of Psychiatry, Addiction Institute of Mount Sinai, New York, United States of America; 6 Psychosomatic Research Center, Isfahan University of Medical Sciences, Isfahan, Iran; 7 Vice President Of Sales, Marketing, Export & Medical Department, Darou Pakhsh Pharmaceutical Mfg.Co., Tehran, Iran; 8 Pharmacovigilance & Clinical Trial Manager, Darou Pakhsh Pharmaceutical Mfg Co., Tehran, Iran; 9 Department Of Health Management And Economics, School of Medicine, AJA University of Medical Sciences, Tehran, Iran; 10 Psychiatry, Institute of Mental Health, Vancouver, Canada; 11 Psychiatric Research Center, Roozbeh Hospital, Department Of Psychiatry, Faculty of Medicine, Tehran University of Medical Sciences, Tehran, Iran

**Keywords:** Iran, Opium tincture, Opioid substitution treatment, Randomized clinical trial

## Abstract

**Introduction:**

Opium tincture (OT) is widely used for opioid substitution treatment (OST) in Iran.

**Objectives:**

To determine if OT is a safe and effective medication for OST.

**Methods:**

Opium Trial was a multicenter, double‐blind, noninferiority randomized controlled trial, with 204 participants with opioid dependence in Iran. Participants were then randomized to OT or methadone arms with an allocation ratio of 1:1 and were followed for 12 weeks. The primary outcome was retention in treatment, compared between the two groups using both intention-To-Treat (ITT) and Per-Protocol (PP) analyses.

**Results:**

A total of 70 participants (IT: 68.6%, PP: 69.3%) in methadone arm and 61 participants (ITT: 59.8%, PP: 60.4%) in OT arm remained in the treatment. The relative retention rate was 1.15 (0.97, 1.36) in both analyses in favour of methadone. A total of 46 out of 152 (30.3%) participants in OT arm and 83 out of 168 (49.4%) participants in methadone arm reported opioid use outside the treatment. The difference in these two proportions (OT - methadone) was 19%: (10%, 28%) in favour of OT. The proportion of patients with adverse events were not different between the two arms (P = 0.06). There was no serious AE in OT arm.

**Conclusions:**

Opium tincture is a clinically effective and safe medication, but this study could not conclude if it was as equally effective as methadone in retaining participants in treatment, but it showed that OT was superior to methadone in reducing opioid use outside the treatment.

**Disclosure:**

No significant relationships.

